# MRI-Based Machine Learning Fusion Models to Distinguish Encephalitis and Gliomas

**DOI:** 10.1007/s10278-023-00957-z

**Published:** 2024-01-12

**Authors:** Fei Zheng, Ping Yin, Li Yang, Yujian Wang, Wenhan Hao, Qi Hao, Xuzhu Chen, Nan Hong

**Affiliations:** 1 Department of Radiology, Peking University People’s Hospital, No. 11 Xizhimen South Street, Xicheng District, Beijing, People’s Republic of China; 2 Imaging Department, Shanxi Province, Shanxi Provincial People’s Hospital, Shanxi Medical University, No. 359 Heping North Road, Jiancaoping District, Taiyuan, People’s Republic of China; 3 Department of Radiology, Fengtai District, Beijing Tiantan Hospital, Capital Medical University, No.119 South Fourth Ring West Road, Beijing, People’s Republic of China

**Keywords:** Encephalitis, Gliomas, Machine learning, Deep learning, Magnetic resonance imaging

## Abstract

This paper aims to compare the performance of the classical machine learning (CML) model and the deep learning (DL) model, and to assess the effectiveness of utilizing fusion radiomics from both CML and DL in distinguishing encephalitis from glioma in atypical cases. We analysed the axial FLAIR images of preoperative MRI in 116 patients pathologically confirmed as gliomas and clinically diagnosed with encephalitis. The 3 CML models (logistic regression (LR), support vector machine (SVM) and multi-layer perceptron (MLP)), 3 DL models (DenseNet 121, ResNet 50 and ResNet 18) and a deep learning radiomic (DLR) model were established, respectively. The area under the receiver operating curve (AUC) and sensitivity, specificity, accuracy, negative predictive value (NPV) and positive predictive value (PPV) were calculated for the training and validation sets. In addition, a deep learning radiomic nomogram (DLRN) and a web calculator were designed as a tool to aid clinical decision-making. The best DL model (ResNet50) consistently outperformed the best CML model (LR). The DLR model had the best predictive performance, with AUC, sensitivity, specificity, accuracy, NPV and PPV of 0.879, 0.929, 0.800, 0.875, 0.867 and 0.889 in the validation sets, respectively. Calibration curve of DLR model shows good agreement between prediction and observation, and the decision curve analysis (DCA) indicated that the DLR model had higher overall net benefit than the other two models (ResNet50 and LR). Meanwhile, the DLRN and web calculator can provide dynamic assessments. Machine learning (ML) models have the potential to non-invasively differentiate between encephalitis and glioma in atypical cases. Furthermore, combining DL and CML techniques could enhance the performance of the ML models.

**Supplementary Information:**

The online version contains supplementary material available at 10.1007/s10278-023-00957-z.

## Introduction

Glioma and encephalitis are prevalent diseases affecting the central nervous system. Surgery is commonly considered as the initial treatment for glioma [1], while non-operative therapy is the primary approach for managing encephalitis [2]. In atypical cases where encephalitis and glioma exhibit very similar manifestations, the laboratory tests are atypical, and the clinical symptoms and signs of these conditions often coincide [3–8]. This diagnostic dilemma can result in unintentional surgery or delayed treatment. Early recognition and prompt initiation of a range of immunotherapies, especially for patients with identifiable antibodies against neuronal cell surface proteins, are crucial for improving the outcomes of those with autoimmune encephalitis (AIE) [9]. Therefore, it is paramount to explore alternative noninvasive diagnostic tools to guide appropriate treatment.

The diagnosis of encephalitis relies on both clinical and paraclinical data, including brain magnetic resonance imaging (MRI). Conventional brain MRI is particularly valuable when the clinical context is uncertain [10]. With current conventional MR imaging methods, differentiating encephalitis from a classical enhancing glioma with perifocal edema, mass effect and necrosis is not challenging. Nevertheless, certain gliomas, particularly lower-grade gliomas that originate from supporting cells in the brain and encompass astrocytomas, oligodendrogliomas or mixed gliomas [11], exhibit focal area enhancement or lesions without enhancement, lacking mass effect or necrosis. This resemblance to encephalitis can lead to misdiagnosis and subsequent treatment delays [8, 12]. Conversely, certain cases of encephalitis present with a noticeable mass effect due to the significant extent, often leading to misdiagnosis as a glioma [13]. There have been multiple published cases of adult brain tumours initially misidentified as encephalitis, such as those documented by Talathi et al. and Wang et al. [7, 14]. Numerous published cases of adult encephalitis initially misdiagnosed as brain tumours have also been reported, including those by Panagopoulos et al. and Halling et al. [5, 15].

Presently, machine learning (ML) is extensively employed in the field of neurological diseases to enhance clinical decision-making. Several studies have demonstrated that ML can distinguish the various pathological subtypes of gliomas [16] and assess the status of molecular and genetic markers associated with the brain tumour [17]. It has been employed to distinguish between glioblastoma and tumefactive demyelinating lesions [18]. These studies suggest that ML proves to be a potent analytical tool in evaluating radiological data related to glioma and encephalitis. To the best of our knowledge, there have been very limited reports on the use of ML based on MRI to distinguish between encephalitis and glioma in atypical cases. In one study, brain inflammation was differentiated from grade II glioma in a cohort of just 57 patients [19]. The other study employed only MR-based deep learning (DL) to differentiate between glioma and encephalitis [20]. The objective of this study was to compare the performance of the classical machine learning (CML) model and the DL model, and assess the effectiveness of utilizing radiomic features extracted from both CML and DL in distinguishing encephalitis from glioma in atypical cases.

## Materials and Methods

This retrospective study was approved by the institutional review boards of the Beijing Tiantan Hospital, Capital Medical University (ID: KY2022-214-02), and the requirement for informed consent was waived.

### Patient Data

In this study, 116 patients (mean age ± standard deviation, 42.3 ± 17.2 years old; 63 men and 53 women) pathologically confirmed as gliomas and clinically diagnosed with encephalitis in our medical institute between January 1, 2019 and March 31, 2023 were recruited. The diagnosis of AIE was based on the 2016 and 2021 diagnostic criteria [10, 21]. The current guidelines for diagnosing AIE are applicable to children as well [22]. Infectious encephalitis diagnosis, on the other hand, required confirmation of an infectious pathogen. Patient clinical data were retrieved and analyzed from electronic medical records. The detailed selection process is shown in Supplementary Fig. S1. The imaging data is restricted to patients of Asian descent due to geographical constraints.

### MRI Acquisition and Segmentation

All patients underwent preoperative head MRI scans, which included in our study is a single FLAIR sequence as it provides the clearest visualization of lesions. For specific MR scanning parameters, please refer to Supplementary Table 1. The raw MRI data were obtained from our institute’s Picture Archiving and Communication System in the format of Digital Imaging and Communications in Medicine (DICOM) and subsequently transferred to a personal computer.

First, the image format was converted from DICOM to NIFTI. Subsequently, all images underwent normalization, with the pixel spacing resampled to 1 × 1 × 0 mm^3^. The image analysis was performed using ITK-SNAP 3.8.0 (http://www.itksnap.org). In this software, the neuroradiologist manually outlined the abnormal hyperintensity on the FLAIR sequence for each slice displaying the lesion. Following the delineation across consecutive slices, the data were saved as volumes of interest (VOIs). The VOIs were delineated by an experienced neuroradiologist (F.Z., with 3 years of neuroradiology experience) and independently confirmed by another neuroradiologist (X.Z.C., with 15 years of neuroradiology experience).

### Study Design

**Fig. 1:**
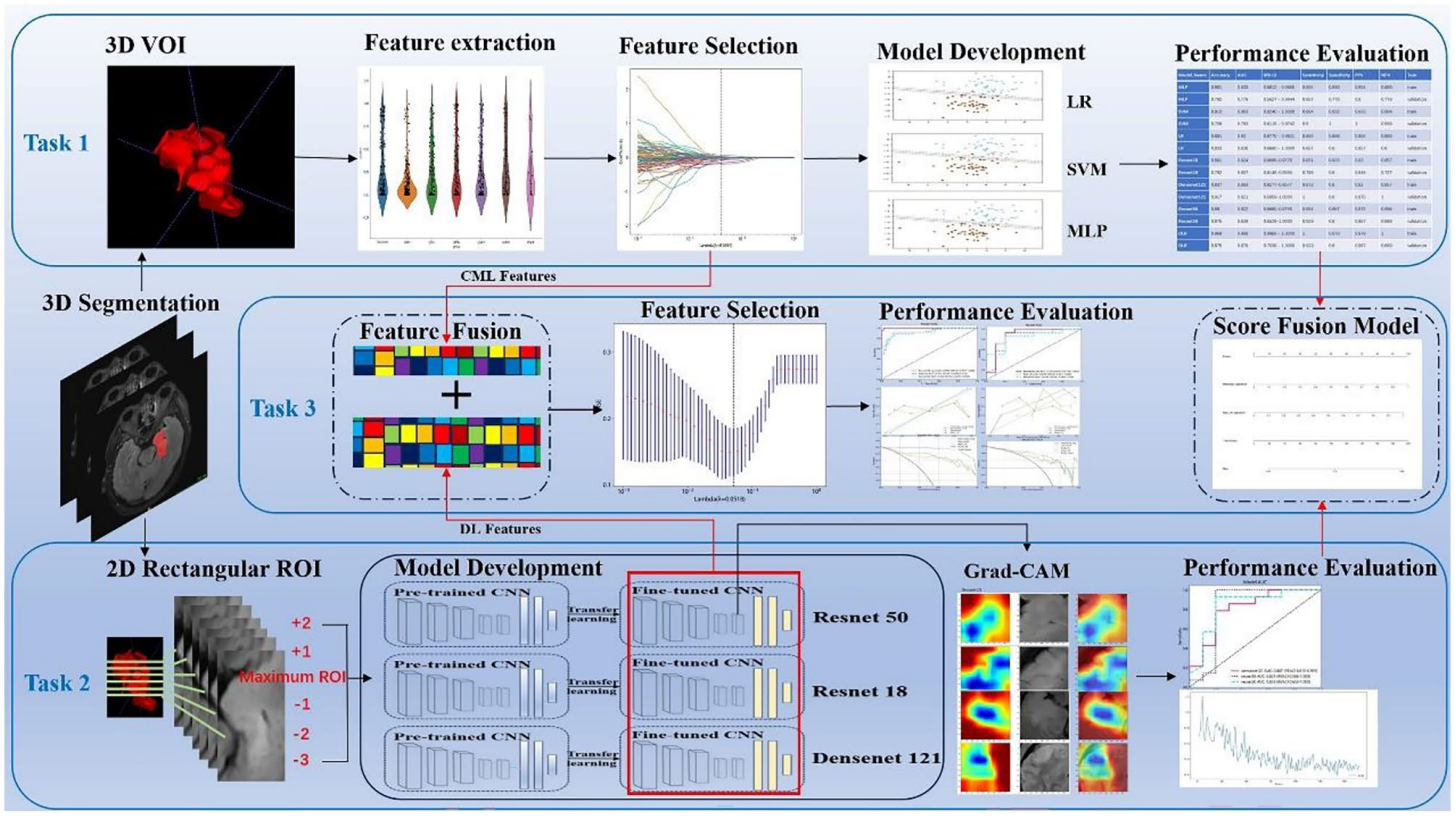
The workflow chart of our study. Including 3 tasks: (1) task 1 consisted of establishing 3 CML models (logistic regression (LR); support vector machine (SVM); and multi-layer perceptron (MLP)) using the FLAIR sequence; (2) task 2 involved constructing 3 DL models (DenseNet 121, ResNet 50 and ResNet 18) based on FLAIR sequence; and (3) task 3 focused on building 2 fusion models, which are feature fusion model and predictive score fusion model

In the current study, we aimed to establish 3 ML models: (1) task 1 consisted of establishing 3 CML models (logistic regression (LR), support vector machine (SVM) and multi-layer perceptron (MLP)) using the FLAIR sequence; (2) task 2 involved constructing 3 DL models (DenseNet 121, ResNet 50 and ResNet 18) based on FLAIR sequence; and (3) task 3 focused on building 2 fusion models, which are feature fusion model and predictive score fusion model. The feature fusion model was based on selecting FLAIR-based CML features and DL features. The features were then combined to create a deep learning radiomic (DLR) model. The predictive score fusion model, a deep learning radiomic nomogram (DLRN), was constructed by combining CML and DL scores using multivariate LR. An online web calculator embedding a dynamic nomogram with binary logistic regression model was also developed. The study workflow is illustrated in Fig. 1.

### Task 1: Construction and Validation of the CML Model

A total of 1015 handcrafted CML features were extracted. Details of the CML features can be found in Supplementary Fig. S2. And the patients were randomly divided into training and internal validation sets in an 8:2 ratio.

To select the most informative radiomic features for subsequent model building, a series of feature selection strategies were implemented. First, the radiomic features were normalized using the *z* score method. Next, the Mann-Whitney *U* test statistical test was performed on all radiomic features, with only those features having a *p* value < 0.05 being retained. For features with high repeatability, Spearman’s rank correlation coefficient was used to calculate the correlation between features; if the correlation coefficient between two features exceeds 0.9, only one of the features was retained. The remaining CML features underwent additional screening using the least absolute shrinkage and selection operator (LASSO) technique. The optimal *λ* was determined through 10-fold cross-validation, where the value providing the minimum cross-validation error was selected.

Following LASSO feature screening, the selected features were input into CML (LR, SVM, MLP) for risk model construction. Default hyperparameters were utilized for all models. In the case of SVM implementation, the penalty relaxation variable C was set to the default value of “1.0”, and the kernel function employed was “rbf”. For LR, the default values of fit_intercept and positive were set to “true” and “false”, respectively. In the case of MLP, the activation function used was “the rectified linear unit”, with a total of three hidden layers consisting of 128, 64 and 32 neurons, respectively. Other default parameters are available at https://scikitlearn.org/stable/modules/generated/sklearn.svm.SVC.html#sklearn.svm.SVC, https://scikitlearn.org/stable/modules/generated/sklearn.linear_model.LinearRegression.html#sklearn.linear_model.LinearRegression and https://scikitlearn.org/stable/modules/generated/sklearn.neural_network.MLPClassifier.html#sklearn.neural_network.MLPClassifier. The area under the receiver operating characteristic (ROC) curve (AUC) served as the evaluation criterion for model performance. The final classifier was then applied to the internal validation sets, and various metrics (sensitivity, specificity, accuracy, negative predictive value (NPV), positive predictive value (PPV) and AUC) were calculated in both the training and validation sets to assess model performance.

### Task 2: Construction and Validation of the DL Model

First, all images underwent conversion from NIFTI to portable network graphics (PNG) format. To capture comprehensive 2.5D signal intensity information from the tumour, the extraction process involved inputting axial FLAIR images and VOI. The axial slice within the smallest rectangular box containing the mask was selected as the “maximum tumour image”. Additionally, five other images were extracted from slices adjacent to the maximum tumour image. These included 1 upper (+ 1), 2 upper (+ 2), 1 lower (− 1), 2 lower (− 2) and 3 lower (− 3) slices within the VOI. When the VOI is too small and the adjacent structure does not have 5 layers, only the layers within the VOI are cropped out. Consequently, a total of six or less axial slices per patient were chosen based on the VOI and treated as individual samples for model development and testing.

The datasets were divided into a training set and an internal validation set using the same splitting strategy adherence to the CML model division. The original image consisted of the image slice showing the maximum tumour region of interest (ROI) area and slices located + 1, + 2, − 1, − 2 and − 3 (totalling to 696 images from 116 patients); this 2.5D approach has demonstrated robust performance compared to 2D or 3D image classification methods, and also achieves significantly lower computational cost [23].

The transfer learning models used in this study were DenseNet 121, ResNet 50 and ResNet 18, all of which were pretrained on the ImageNet dataset to initialize the weight values. Prior to training, the input 2D rectangular ROIs were resized to dimensions of 224 × 224 pixels for the DL models. The size of the fully connected layers was adjusted from 1000 to 2 to enable the binary classification of patients into glioma and encephalitis groups. Model training involved forward computation and backpropagation. The network weights were updated using a cross-entropy loss function for the predictive task. In this study, the models were trained using an adaptive moment estimation optimizer with batch size of 32. We utilized the “torch.Optim.Lr_scheduler.CosineAnnealingLR” library provided by PyTorch 1.8.0 to dynamically adjust the learning rate. The initial learning rate was set to 0.01. As the number of training epochs increases, the learning rate gradually decreases. The average loss value in the training set is computed every five epochs. If the decrease in loss value is less than 5% compared to the previous cycle, the program determines that the training process is complete. ResNet 18, ResNet 50 and DenseNet 121 were trained for 50, 55 and 30 epochs, respectively. More information about working mode is available at https://github.com/pytorch/vision/torchvision/models. The performances of the DL models were also assessed using sensitivity, specificity, accuracy, NPV, PPV and AUC. An illustration showcasing the Resnet network architectures can be accessed in Supplementary Fig. S3.

### Task 3: Development of the DLR models and the DLRN

Once construction and validation of the DL models were completed, the network parameters were fixed, and the fixed models were used as a feature extractor. The DL features were extracted from the penultimate layer of the fine-tuned network for each patient in the training and validation sets. To enhance the transparency of the model’s decision-making process and to investigate its interpretability, gradient-weighted class activation mapping (Grad-CAM) was employed to visualize the models. The gradient information from the last convolutional layer of the networks was used for weighted fusion to generate a class activation map that highlighted the important regions of the target classification image [24].

DL models extract a multitude of features, making it necessary to employ dimension reduction techniques such as principal component analysis (PCA) to effectively handle the high dimensionality of the extracted features. The number of features is drastically reduced through PCA. Subsequently, these features were combined with CML features for further DLR modelling. The feature screening methods and model building process for the DLR model mirrored those utilized for the CML model. The integration of DL features and CML features aimed to maximize their respective characteristics and overcome instability caused by the limited sample size. The performance of the best CML model, the best DL model and the DLR model was assessed using the AUC with 95% confidence interval (CI). To investigate the net benefit of the discrimination model across the entire range of probability thresholds, we employed decision curve analysis (DCA) [25, 26]. The agreement between the predicted and actual outcomes of the model was evaluated using calibration curve. Calibration curve that closely aligns with the 45° diagonal indicates a higher level of model accuracy [27]. DCA and calibration curves were performed to evaluate the clinical utility of the three models.

Additionally, a predictive score fusion model was also established to construct the DLRN. The DLRN was constructed by combining the respective CML and DL scores utilizing LR. It can be calculated for each patient in both the training and test sets by combining the DL and CML scores, weighted by their respective coefficients. A web-based calculator was also developed to compute the correlation between the screening variables (CML and DL scores) and the estimated probabilities of encephalitis.

### Statistical Analysis

Differences in clinical characteristics between the training and validation sets were evaluated using the *t* test and chi-squared test. The analysis was conducted with statistical software SPSS 26 (SPSS Inc, Armonk, NY), and statistical significance was defined as a *p* value < 0.05.

## Results

### Clinical Characteristics

The patients were randomly allocated to two sets: training (*n* = 92) and validation (*n* = 24), with the mean ages of 42.61 and 41.33 years, respectively. No significant difference was observed in age and gender between the two sets of patients. Detailed clinical characteristics of patients are listed in Table 1**.**

**Table 1:** Baseline characteristics of patients in cohorts

Feature name	Train-All	Train-Glioma	Train-Encephalitis	*P* value	Test-All	Test-Glioma	Test-Encephalitis	*P* Value
Age	42.61 ± 16.74	45.04 ± 14.56	40.28 ± 18.45	0.17	41.33 ± 19.10	41.30 ± 17.46	41.36 ± 20.84	0.99
Gender				0.69				1.00
Female	42 (45.65)	22 (48.89)	20 (42.55)		11 (45.83)	5 (50.00)	6 (42.86)	
Male	50 (54.35)	23 (51.11)	27 (57.45)		13 (54.17)	5 (50.00)	8 (57.14)	

### Task 1: CML Model Construction and Validation

**Fig. 2:**
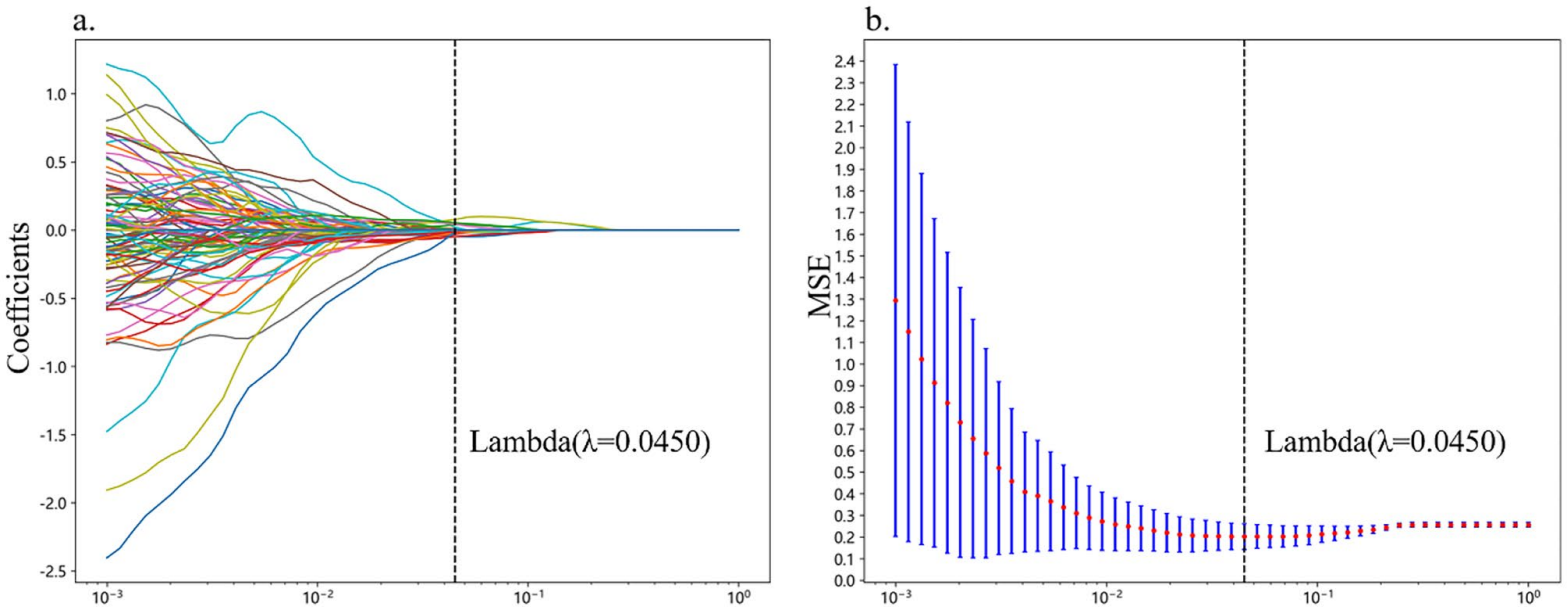
Coefficients of 10-fold cross-validation in CML model (**a**). MSE of 10-fold cross-validation in CML model (**b**)

**Fig. 3:**
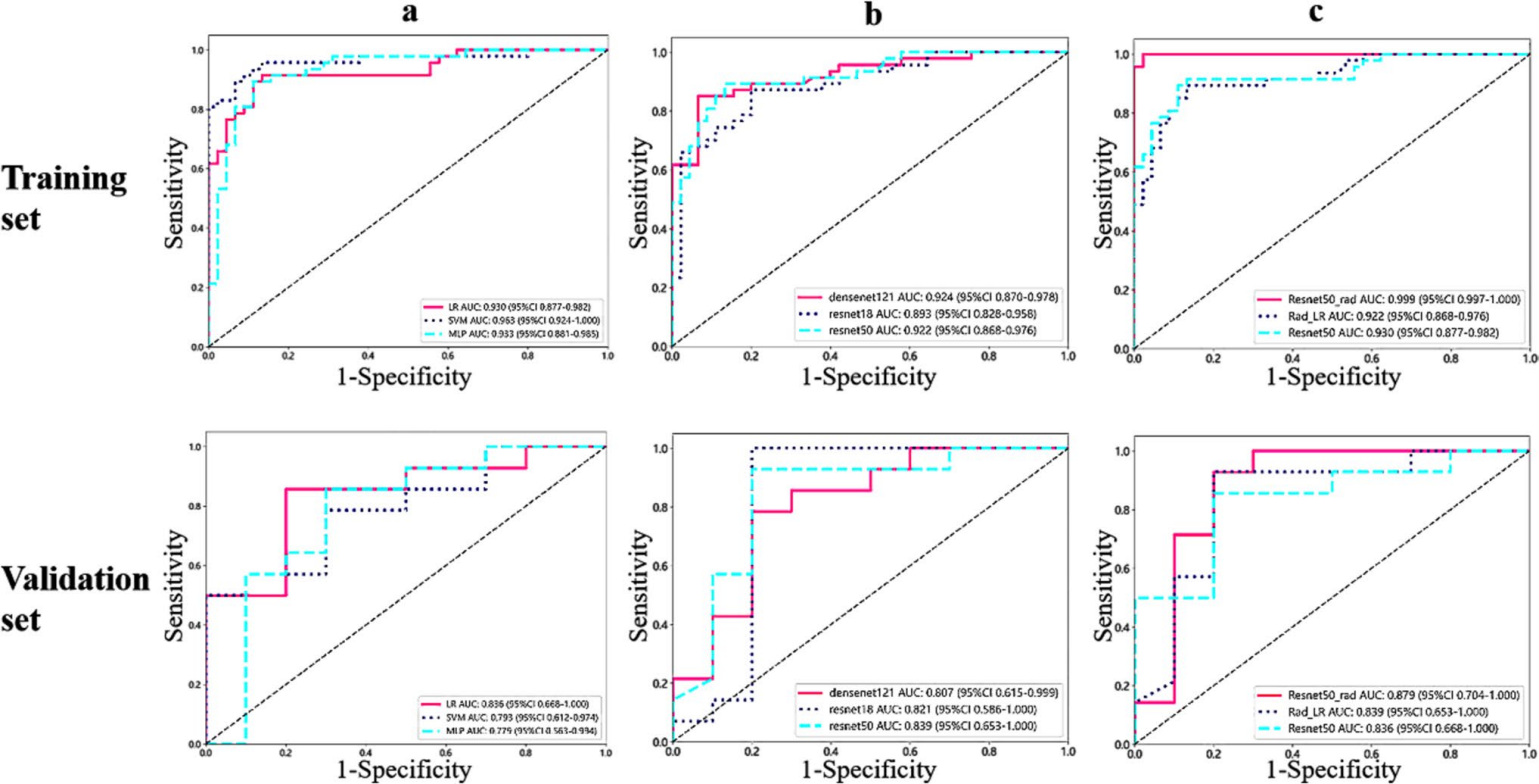
**a** ROC analysis of different CML models; **b** ROC analysis of different DL models; and **c** the AUCs of the best CML model, the best DL model and the DLR model on the training and validation cohort

A total of 7 categories, 1015 handcrafted CML features are extracted, including 198 first-order features, 14 shape features and the remained texture features. All handcrafted features are extracted with an in-house feature analysis program implemented in Pyradiomics (http://pyradiomics.readthedocs.io). The extracted features and their corresponding *p* value results are presented in Supplementary Fig. S4. After performing the Mann-Whitney *U* test and calculating Spearman’s rank correlation coefficient, nonzero coefficients were chosen to construct the Rad score using a LASSO logistic regression model. The coefficients and mean standard error (MSE) from 10-fold validation are presented in Fig. 2. And Supplementary Fig. S5 shows the coefficient value in the final selected none zero features. Table 2 displays the performance of the CML model utilized for distinguishing encephalitis from gliomas, with the LR model performing the best compared with the SVM and the MLP classifier. The LR model exhibited the highest AUC values of 0.930 and 0.836 on the training and validation cohorts, respectively. Figure 3 illustrates the AUC of each CML model on both the training and validation cohorts. Furthermore, Supplementary Fig. S6. displays the confusion matrices of the prediction results and presents the DCA of each model.

**Table 2:** The performance of each model in training and validation sets

Model type	Model name	AUC	95% CI	Accuracy	Sensitivity	Specificity	PPV	NPV	Task
CML model	MLP	0.933	0.8812–0.9855	0.891	0.894	0.889	0.894	0.889	Train
	MLP	0.779	0.5627–0.9944	0.792	0.857	0.778	0.800	0.778	Validation
	SVM	0.963	0.9240–1.0000	0.913	0.894	0.933	0.933	0.894	Train
	SVM	0.793	0.6115–0.9742	0.708	0.500	1.000	1.000	0.588	Validation
	LR	0.930	0.8770–0.9821	0.891	0.894	0.889	0.894	0.889	Train
	LR	**0.836**	0.6680–1.0000	0.833	0.857	0.800	0.857	0.800	Validation
DL model	Resnet18	0.924	0.8695–0.9778	0.891	0.851	0.933	0.930	0.857	Train
	Resnet18	0.807	0.6149–0.9994	0.792	0.786	0.800	0.846	0.727	Validation
	Densenet121	0.893	0.8277–0.9577	0.837	0.872	0.800	0.820	0.857	Train
	Densenet121	0.821	0.5859–1.0000	0.917	1.000	0.800	0.875	1.000	Validation
	Resnet50	0.922	0.8680–0.9755	0.880	0.894	0.867	0.875	0.886	Train
	Resnet50	**0.839**	0.6529–1.0000	0.875	0.929	0.800	0.867	0.889	Validation
DLR model	DLR	0.999	0.9968–1.0000	0.989	1.000	0.978	0.979	1.000	Train
	DLR	**0.879**	0.7038–1.0000	0.875	0.929	0.800	0.867	0.889	Validation

### Task 2: DL Model Construction and Validation

**Fig. 4:**
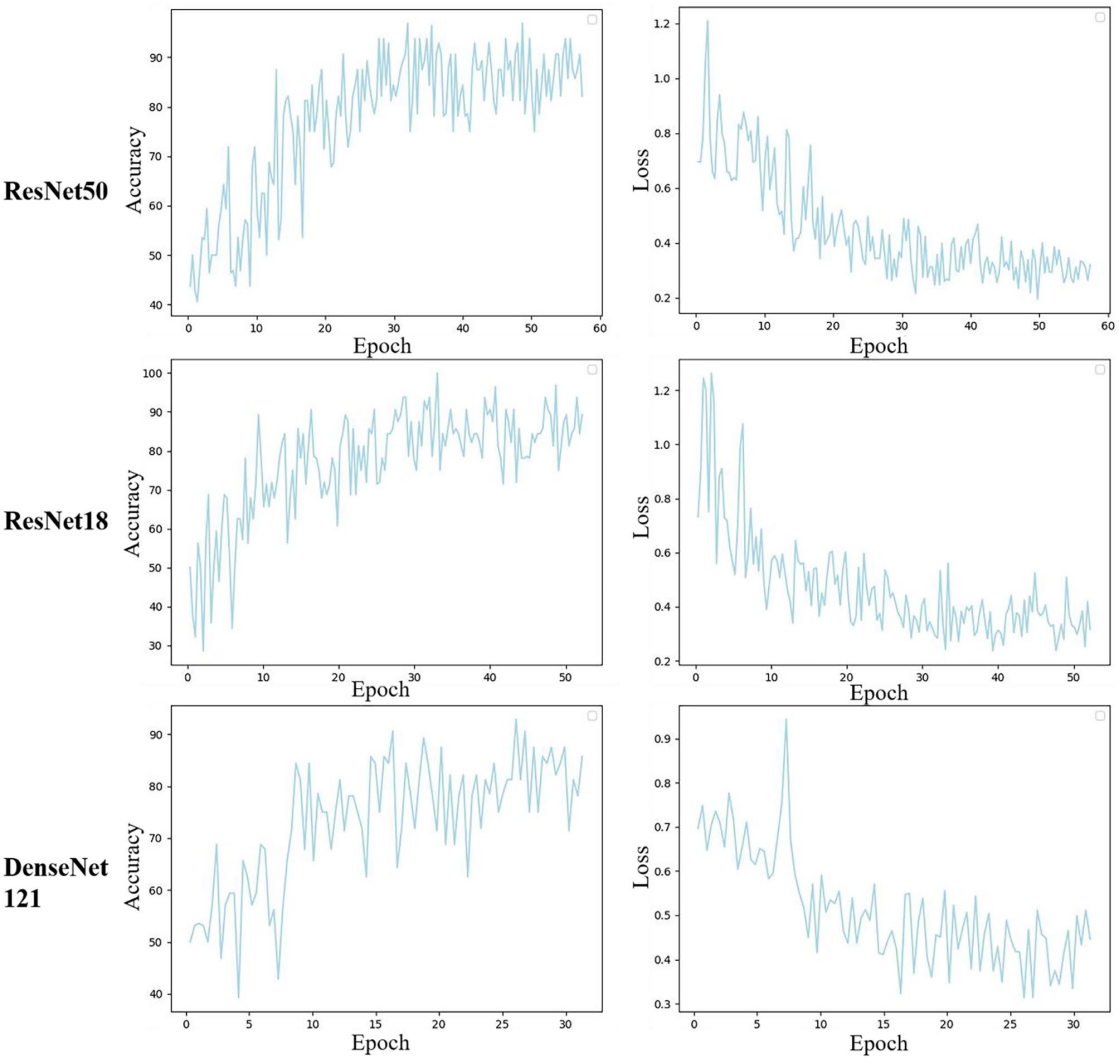
The accuracy rate and loss values of various deep learning models in the training set varied as the epoch progressed. ResNet50 demonstrated the lowest loss value, indicating better error learning during training, and achieved faster convergence compared to ResNet18 and DenseNet 121

The ResNet50 model exhibited superior performance compared to the other two DL models in the validation set (Table 2 and Fig. 3). In the validation set, the ResNet50 model demonstrated the highest classification performance, achieving an AUC of 0.839, accuracy of 0.875, sensitivity of 0.929, specificity of 0.800, PPV of 0.867 and NPV of 0.889. Moreover, ResNet50 consistently outperformed the LR model, exhibiting an AUC of 0.836, accuracy of 0.833, sensitivity of 0.857, specificity of 0.800, PPV of 0.857 and NPV of 0.800. ResNet50 demonstrated the lowest loss value, indicating better error learning during training [28], and achieved faster convergence compared to the other two DL models (Fig. 4).

### Task 3: Development of the DLR Models and the DLRN

**Fig. 5:**
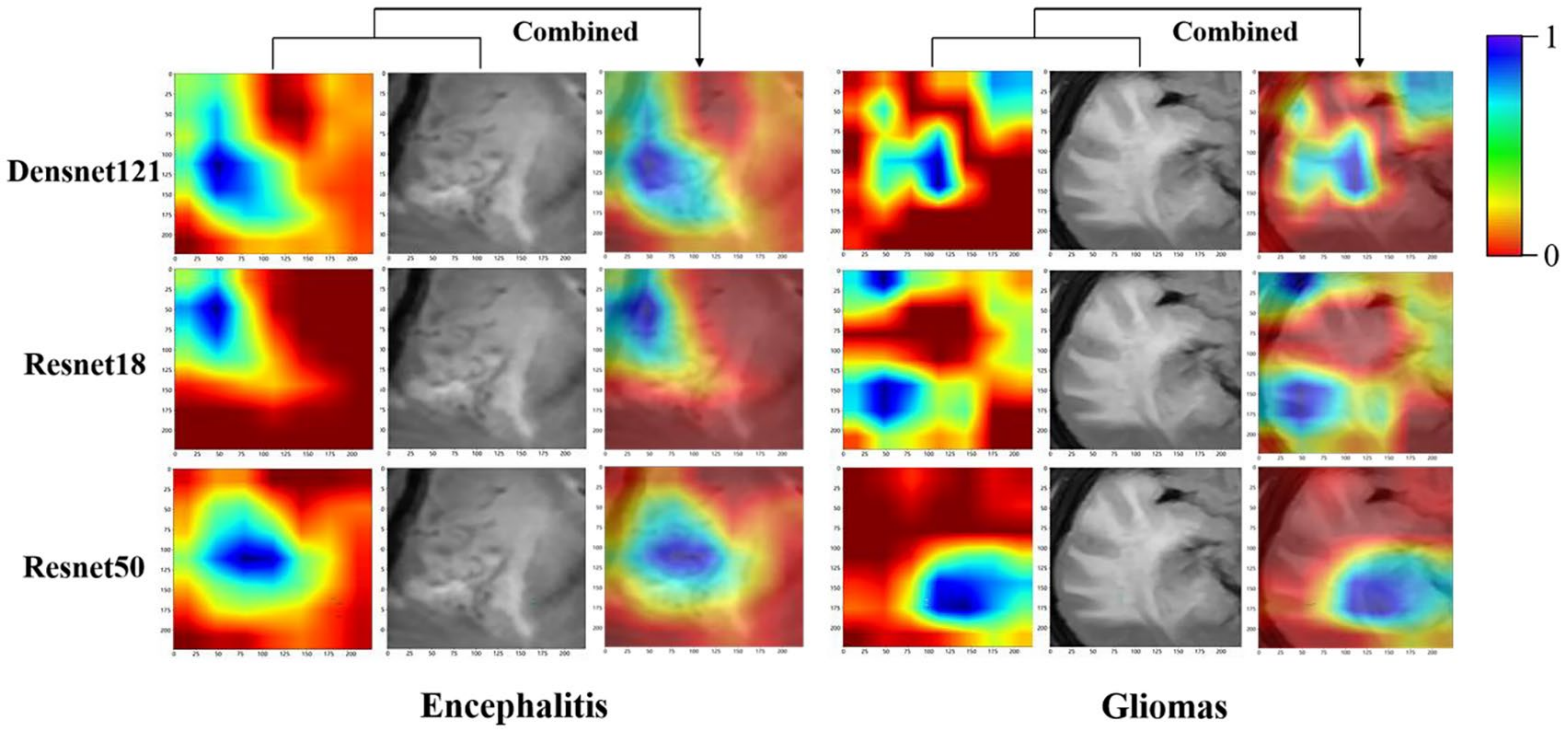
The attention regions of different DL models in gliomas and encephalitis on FLAIR image, which are heat maps showing the areas of the image that the DL models focus on for their decision-making process. The scale bar from red to blue indicates the increased contribution of the location to the model’s classification

Considering the superior predictive performance of the resnet50 model, the DL features were extracted from the fixed resnet50 model. Each PNG image was used to extract a total of 2049 DL features. From task 2, each patient contributed 6 PNG images, resulting in a total of 12,294 DL features for each patient. Figure 5 presents the Grad-CAM representations, which are heat maps showing the areas of the image that the DL models focus on for their decision-making process. The scale bar from red to blue indicates the increased contribution of the location to the model’s classification. In terms of model interpretability, ResNet50 exhibited distinct attention regions, predominantly concentrating on internal regions of the tumour that align with the radiologist’s areas of concern. Conversely, it displayed limited activation in the boundary regions of the tumour and the tumour regions adjacent to normal brain tissue.

Then utilizing PCA for dimensionality reduction, we extracted 32 DL features from each PNG image. With each patient contributing 6 PNG images, a total of 192 DL features were obtained. PCA is a statistical technique used to simplify and interpret a high-dimensional dataset by identifying the patterns and relationships among variables [29]. Using PCA, the number of DL features was reduced from 12,294 to 192. PCA is not employed for CML since the superiority of CML over DL lies in the presence of screening features with specific formula and definition, and applying PCA for dimensional reduction would eliminate these distinctive advantages [30]. These DL features were then combined with 13 CML features from task 1. In total, 205 DL and CML features were selected, out of which only 22 features remained after employing a LASSO logistic regression model. The coefficients, MSE, coefficient values and Rad score from 10-fold validation are provided in Supplementary Fig. S7 and Supplementary information. Finally, a DLR model was constructed using LR classifier due to its excellent performance in task 1.

Table 2 presents all the models that were utilized for distinguishing encephalitis from gliomas, and it is observed that the DLR model exhibited the highest performance. The DLR model, which is considered the optimal model, demonstrated the highest AUC values on both the training and validation cohorts, reaching 0.999 and 0.879 respectively. Figure 3 illustrates the AUCs of the best CML model (LR), the best DL model (ResNet50) and the DLR model on the training and validation cohort.

**Fig. 6:**
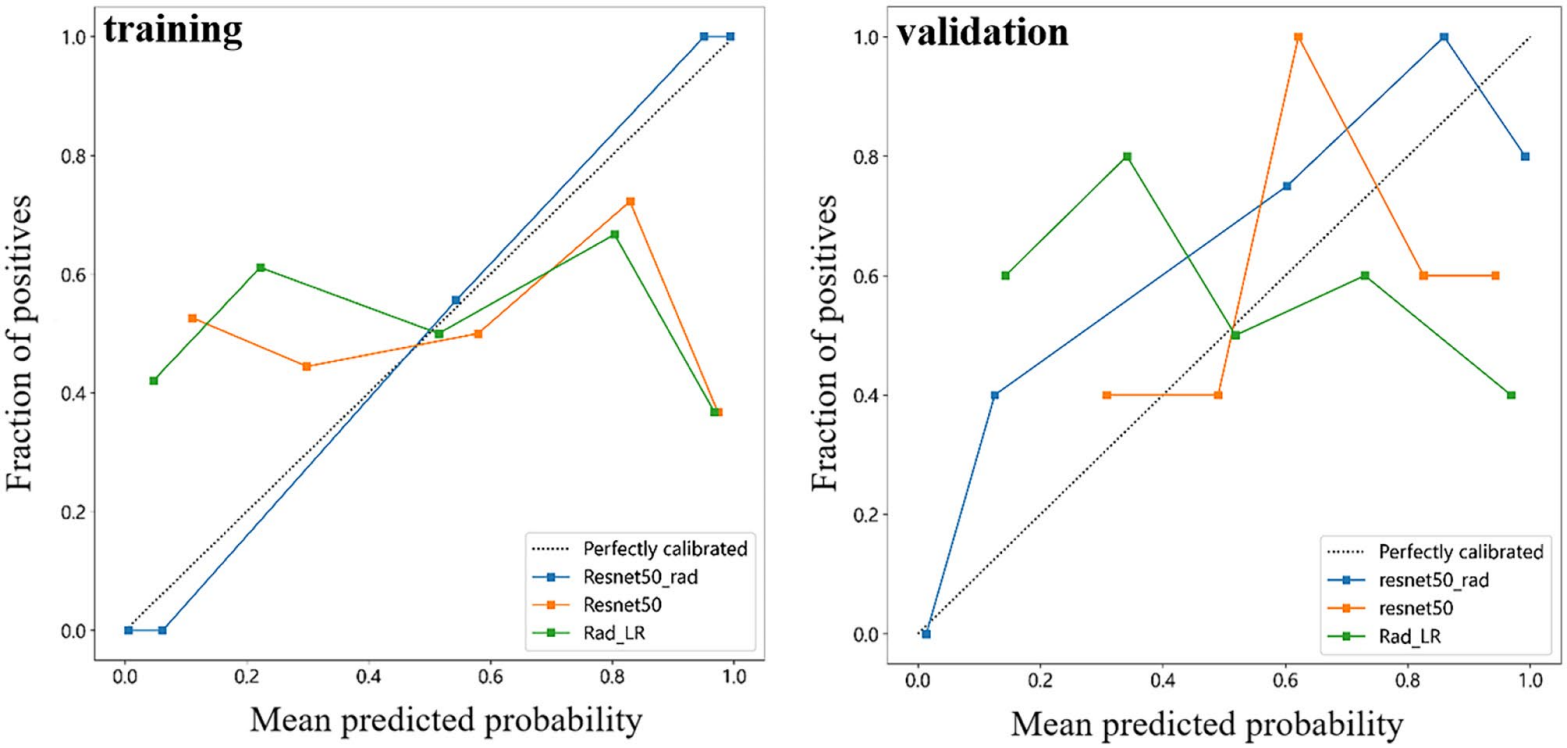
The calibration curves of the best CML, best DL model and DLR model in the training and validation cohort to assess the agreement between the predicted and actual outcomes of the model

**Fig. 7:**
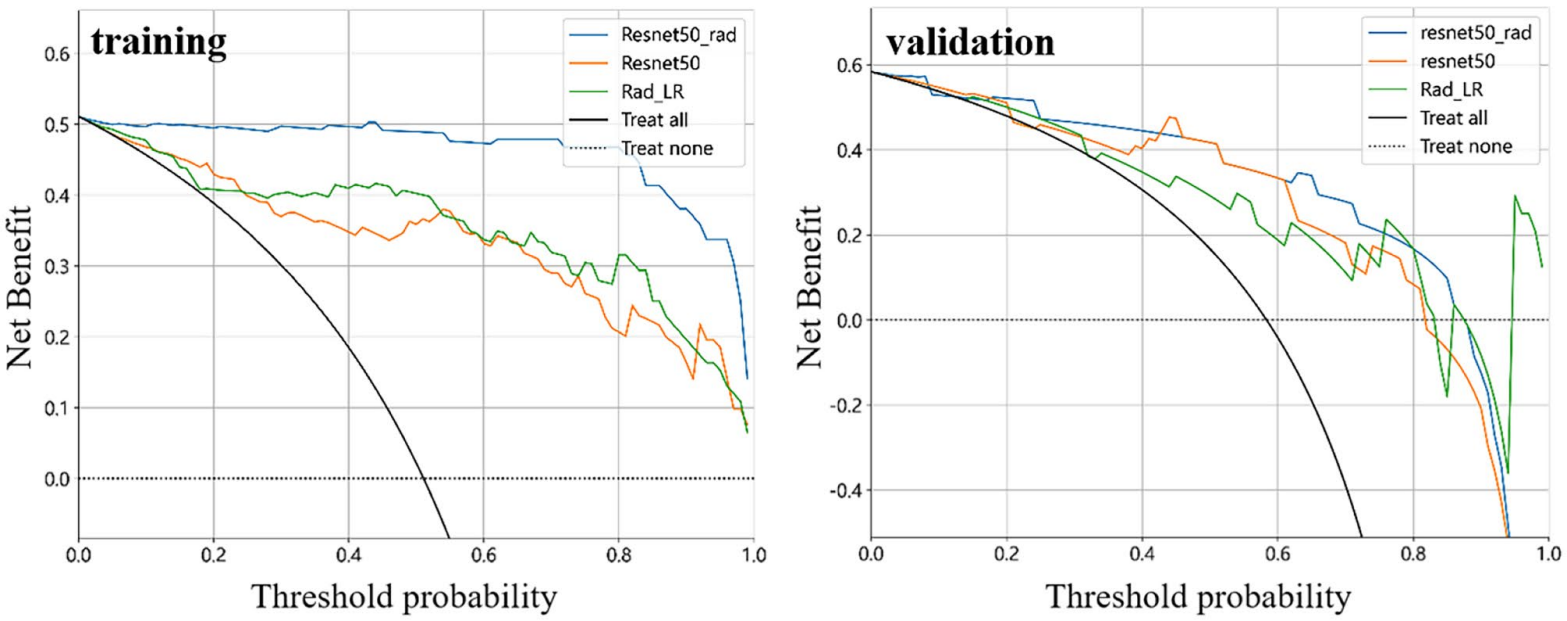
The DCA of the best CML, best DL model and DLR model in the training and validation cohort, demonstrating the net benefit of the discrimination models across the entire range of probability thresholds

In addition, the calibration and DCA of the best CML, best DL model and DLR model are shown in Figs. 6 and 7. Figure 6 shows good agreement between prediction and observation in the validation cohort. Figure 7 demonstrates that the DLR model shows a higher net benefit at all threshold possibilities during training compared to the best CML and the best DL model. Preoperative differentiation between encephalitis and gliomas using DLR model has been shown a better clinical benefit.

**Fig. 8:**
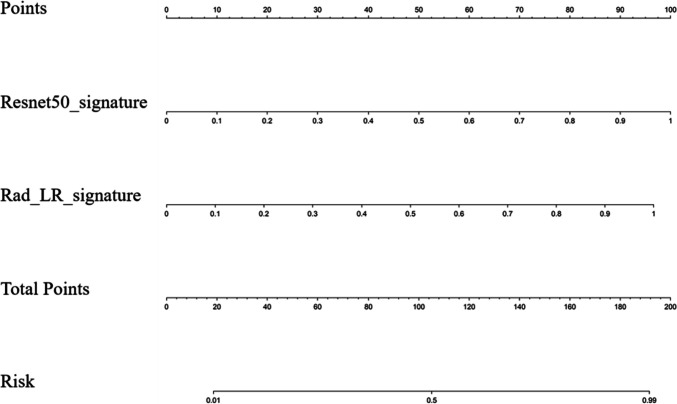
Nomogram for predicting the probability of encephalitis. The values of predictors (ResNet50 signature and LR signature) which were mapped to the points axis can be transformed into risk points. Then, the sum of risk points of predictors in the total points axis can be mapped to the risk axis to obtain the probability of encephalitis

Meanwhile, we developed a predictive score fusion model for constructing the DLRN. Figure 8 depicts how the CML and DL scores are combined through multivariate LR, serving as the foundation for the DLRN architecture. Variable values (ResNet50 signature and LR signature) for individual were determined based on the top Points scale, and subsequently, the points for each variable were summed. Finally, a customized probability was obtained using the bottom Total Points scale. An interactive web calculator, incorporating the dynamic nomogram, was also developed and can be accessed at https://nomogramzf.shinyapps.io/dynnomapp/. An interactive web calculator can provide an accurate prediction probability of encephalitis with 95% confidence interval, enhance the visual representation of the nomogram and improve its clinical usability. By completing the required online form, users will be provided with a personalized predicting probability of encephalitis. Additionally, to compare the best CML model, the best DL model and the DLRN model, Delong test was used. The results of Delong test are shown in the Supplementary Table 2.

## Discussion

In our study, ML models were developed based on the FLAIR sequence and their performance was compared. Regarding the CML models based on the FLAIR sequence, the LR model exhibited the highest performance while the MLP model showed the lowest performance. As for the DL models based on the FLAIR sequence, the Resnet 50 classifier demonstrated the highest performance whereas the Resnet 18 classifier exhibited the lowest performance. The performance disparity among different DL models can be attributed to their diverse internal architectures [31]. DL models outperform CML models, possibly due to the fact that DL enables end-to-end classification and prediction by automatically learning complex features directly from the raw pixels of input images, thus eliminating the need for manually designed hard-coded feature extraction [32, 33]. Importantly, the DLR model demonstrated superior performance compared to the other 2 models. We hypothesize that combining CML parameters with DL parameters can enhance the extraction of valuable information from conventional MRI brain images and improve prediction results, consistent with previous study [34]. In conclusion, our findings suggest that ML models have the potential to non-invasively differentiate between encephalitis and glioma in atypical cases. Furthermore, combining DL and CML techniques could enhance the performance of the ML models.

Our study is based on single FLAIR sequences for two reasons. On the one hand, the cortical hypersignal of encephalitis is most evident on MRI FLAIR sequence [35]. Additionally, FLAIR hyperintensities persist for several weeks longer than on other sequences [36]. On the other hand, each of the other sequences has its own specific defects. In patients with encephalitis, only few cases showed contrast enhancement on contrast-enhanced T1-weighted images (T1WIs) [37]. As for T1WI and T2-weighted images (T2WIs), the lesion exhibits only mild hypo-intensity and hyper-intensity, which makes delineating the lesions challenging. Furthermore, we did not include clinical factors in our study due to the limited number we obtained and their lack of statistical significance, which is consistent with previous studies [19].

Both encephalitis and glioma can present as lesions with mass effect and demonstrate hypo-intensity on T1WI, hyperintensity on T2WI and no enhancement on post-contrast T1WI, leading to similar findings on conventional MR sequences in atypical cases. Magnetic resonance spectroscopy usually detected increased choline concentration and a moderate decrease in NAA concentration in the substance of the encephalitis. These measurements also suggested compatibility with a low-grade lesion, such as astrocytoma [5]. Despite the use of various functional MR techniques for differential diagnosis, there is currently no established expert consensus [7, 38–40]. While one study has reported that conventional MRI features can assist in distinguishing inflammatory lesions from glioma [41], the subjective nature of feature evaluation and the absence of quantitative indicators hinder its clinical utility. A previous study revealed that the two radiologists, despite having 10 and 8 years of experience in diagnosis of central nervous system diseases, achieved an accuracy of only 0.544 and 0.526 respectively for the definite diagnosis [19]. Currently, there is still a diagnostic dilemma in distinguishing encephalitis from glioma in atypical cases using MR imaging. Two typical examples are provided in Supplementary Fig. S8.

Our study expands the work of several recent studies that have focused on differentiation between encephalitis and glioma in atypical cases. In previous research, radiomic analyses were conducted using T1WI and T2WI on a cohort of 57 patients due to the low incidence of atypical cases [19]. In our study, we extended the analysis to include a new sequence and a larger cohort of 116 patients. Additionally, we performed comprehensive radiomic analyses not only on the DL model but also on the CML and fusion models, setting ourselves apart from a prior study that solely utilized DL models (Alexnet, ResNet 50 and Inception-V3) [20]. We conducted a comparison between our model and the Alexnet model as well as the Inception-V3 model to enhance persuasiveness in our task 2. The results clearly indicate that our model outperforms the Alexnet model and the Inception-V3 model in terms of performance. And the corresponding results are presented in Supplementary Fig. S9. The fusion model provides a valuable reference for future studies. The feature fusion approach allows us to leverage the strengths of both CML and DL techniques. The fusion of scores provides an additional level of confidence in the results. By incorporating multiple models and fusion techniques, our study aims to improve the accuracy and reliability of distinguishing between encephalitis and glioma in atypical cases. This research has the potential to greatly benefit future studies in this field.

The current study has several limitations. Firstly, our study relied on retrospectively collected data, and a prospective study is necessary to validate our findings. Second, the sample size from a single-centre study was relatively small. Consequently, multicenter datasets and a larger patient cohort are required to validate the current findings. Third, we solely focused on distinguishing between encephalitis and glioma in atypical cases, without further subtyping, such as AIE or infectious encephalitis. Investigating these aspects will be a vital direction for our future research. In addition, our results do not represent the average of multiple iterations conducted with different random states or seeds. In our forthcoming research, we will compute the average of the outcomes under diverse conditions of random seeds to augment the reliability of the findings. Finally, the web calculator does not accept images and only accepts input of specific values, which limits its utility at present [42, 43]. Our next step is to build software or toolkit that generates prediction probabilities by uploading raw medical images and raw clinical data with one click.

In conclusion, our findings demonstrate the potential utility of ML based on FLAIR for distinguishing atypical cases of encephalitis and glioma which suggests its potential application in assisting clinical decision-making is noteworthy.

### Supplementary Information

Below is the link to the electronic supplementary material.

Supplementary file1 (DOCX 3.13 MB)

## Data Availability

The datasets generated or analyzed during the study are available from the corresponding author on reasonable request.

## Abbreviation

AIE: Autoimmune encephalitis
AUC: Area under the receiver operating curve
CI: Confidence interval
CML: Classical machine learning
DCA: Decision curve analysis
DICOM: Digital imaging and communications in medicine
DL: Deep learning
DLR: Deep learning radiomics
DLRN: Deep learning radiomic nomogram
Grad-CAM: Gradient-weighted class activation mapping
LASSO: Least absolute shrinkage and selection operator
LR: Logistic regression
ML: Machine learning
MLP: Multi-layer perceptron
MRI: Magnetic resonance imaging
MSE: Mean standard error
NPV: Negative predictive value
PCA: Principal component analysis
PNG: Portable network graphics
PPV: Positive predictive value
ROC: Receiver operating characteristic
ROI: Region of interest
SVM: Support vector machine
T1WI: T1-weighted images
T2WI: T2-weighted images
VOI: Volumes of interest
