# Supplementary material for: MRI-Based Machine Learning Fusion Models to Distinguish Encephalitis and Gliomas
Source: J Imaging Inform Med. 2024 Jan 12;37(2):653–65. doi: 10.1007/s10278-023-00957-z (PMC11031538; doi:10.1007/s10278-023-00957-z)
Supplement: Supplementary file 1 — Supplementary file1 (DOCX 3.13 MB) [file 10278_2023_957_MOESM1_ESM.docx]

**Title:** MRI-Based Machine Learning Fusion Models to Distinguish Encephalitis and Gliomas

**Supplementary Material**

**List of Supplementary Material**

Supplementary Methods and Results

Supplementary Fig S1: The flow chart of the enrollment for patients with gliomas or encephalitis.

Supplementary Fig S2: Number and ratio of handcrafted features.

Supplementary Fig S3: An illustration showcasing the Resnet network architectures

Supplementary Fig S4: Statistics of radiomic features.

Supplementary Fig S5: The histogram of the Rad-score based on the selected features.

Supplementary Fig S6: The confusion matrices of the prediction results and the DCA on single CML model.

Supplementary Fig S7: Coefficients, MSE of 10-fold cross validation and the histogram of the Rad-score based on the selected features in DLR model.

Supplementary Fig S8: Two characteristic examples of encephalitis and gliomas in atypical cases are presented.

Supplementary Fig S9: 9a, b demonstrates ROC analysis of different DL models in the training and test sets; 9c, d illustrates the DCA analysis of different DL models the training and test sets.

Supplementary Table 1. The scanning parameters for the axial FLAIR sequence of each MRI scanner.

Supplementary Table 2. The results of Delong test between the best CML, best DL model and DLR model in the training and validation cohort.

Supplementary Reference

**Supplementary Methods and Results**

**Patient Data**

The patients with glioma included in this study were confirmed through pathological examination. For patients who were eventually diagnosed with encephalitis, the patients had to meet the following criteria: presence of encephalitis lesions on MRI, corresponding clinical symptoms, and confirmation through cerebrospinal fluid analysis, antibody testing, virus examination, surgery or pathological biopsy, or observation of complete or substantial disappearance of the lesion or development of encephalomalacia during the follow-up period. Antibodies were detected using a cell-based indirect immunofluorescence assay, which involved analyzing serum, cerebrospinal fluid, or both[1].

**Radiomics score in CML model and DLR model**

After performing the Mann-Whitney U test and calculating Spearman's rank correlation coefficient, nonzero coefficients were chosen to construct the Rad-score using a LASSO logistic regression model in the both CML model and DLR model. The non-zero coefficients of the filtered features were calculated as the respective radiomics score for both CML model and DLR model.

The Rad score of CML model is show as follows:

label = 0.5345144236755537 + +0.024465 * original_shape_Maximum3DDiameter -0.049616 * original_shape_Sphericity -0.082160 * wavelet_HHL_firstorder_Kurtosis -0.039498 * wavelet_HLH_gldm_LargeDependenceHighGrayLevelEmphasis +0.069429 * wavelet_HLL_firstorder_RootMeanSquared +0.030882 * wavelet_HLL_glszm_ZonePercentage +0.016186 * wavelet_LHH_firstorder_RootMeanSquared -0.021704 * wavelet_LHH_glszm_LargeAreaLowGrayLevelEmphasis +0.091688 * wavelet_LHH_glszm_ZonePercentage +0.021142 * wavelet_LHL_glszm_LargeAreaHighGrayLevelEmphasis -0.009912 * wavelet_LLH_glszm_LargeAreaLowGrayLevelEmphasis +0.025610 * wavelet_LLL_glcm_ClusterShade -0.009943 * wavelet_LLL_glcm_Correlation

And Rad score of DLR model is show as follows:

label = 0.5108695652173911 -0.003932 * resnet50original_shape_Sphericity -0.001166 * resnet50wavelet_HLH_gldm_LargeDependenceHighGrayLevelEmphasis +0.031154 * resnet50wavelet_HLL_firstorder_RootMeanSquared +0.029986 * resnet50wavelet_LHH_glszm_ZonePercentage +0.046922 * resnet50wavelet_LLL_glcm_ClusterShade -0.001495 * resnet50wavelet_LLL_glcm_Correlation -0.054225 * resnet50DL_6_3_1 +0.041532 * resnet50DL_1_2_1 -0.016501 * resnet50DL_9_2_1 -0.005757 * resnet50DL_4_2 +0.029184 * resnet50DL_31_2 -0.013261 * resnet50DL_3_1_1 +0.054277 * resnet50DL_4_1_1 +0.004195 * resnet50DL_23_1_1 +0.013487 * resnet50DL_1_1 -0.007544 * resnet50DL_8_1 +0.183867 * resnet50DL_0 -0.125807 * resnet50DL_1 +0.032417 * resnet50DL_3 +0.019208 * resnet50DL_8 +0.009469 * resnet50DL_11 -0.004099 * resnet50DL_21


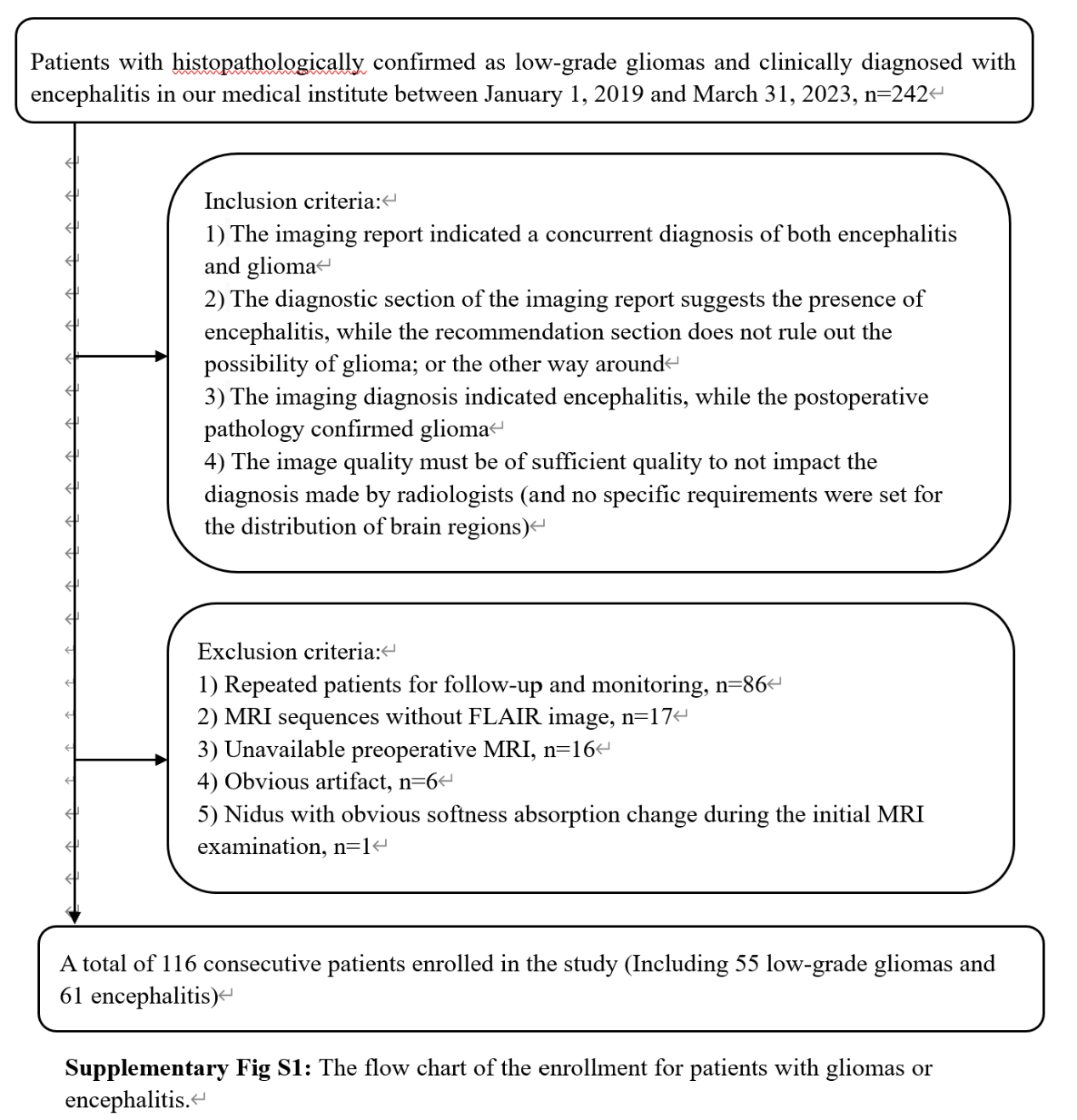


**Supplementary Fig S1:** The flow chart of the enrollment for patients with gliomas or encephalitis.


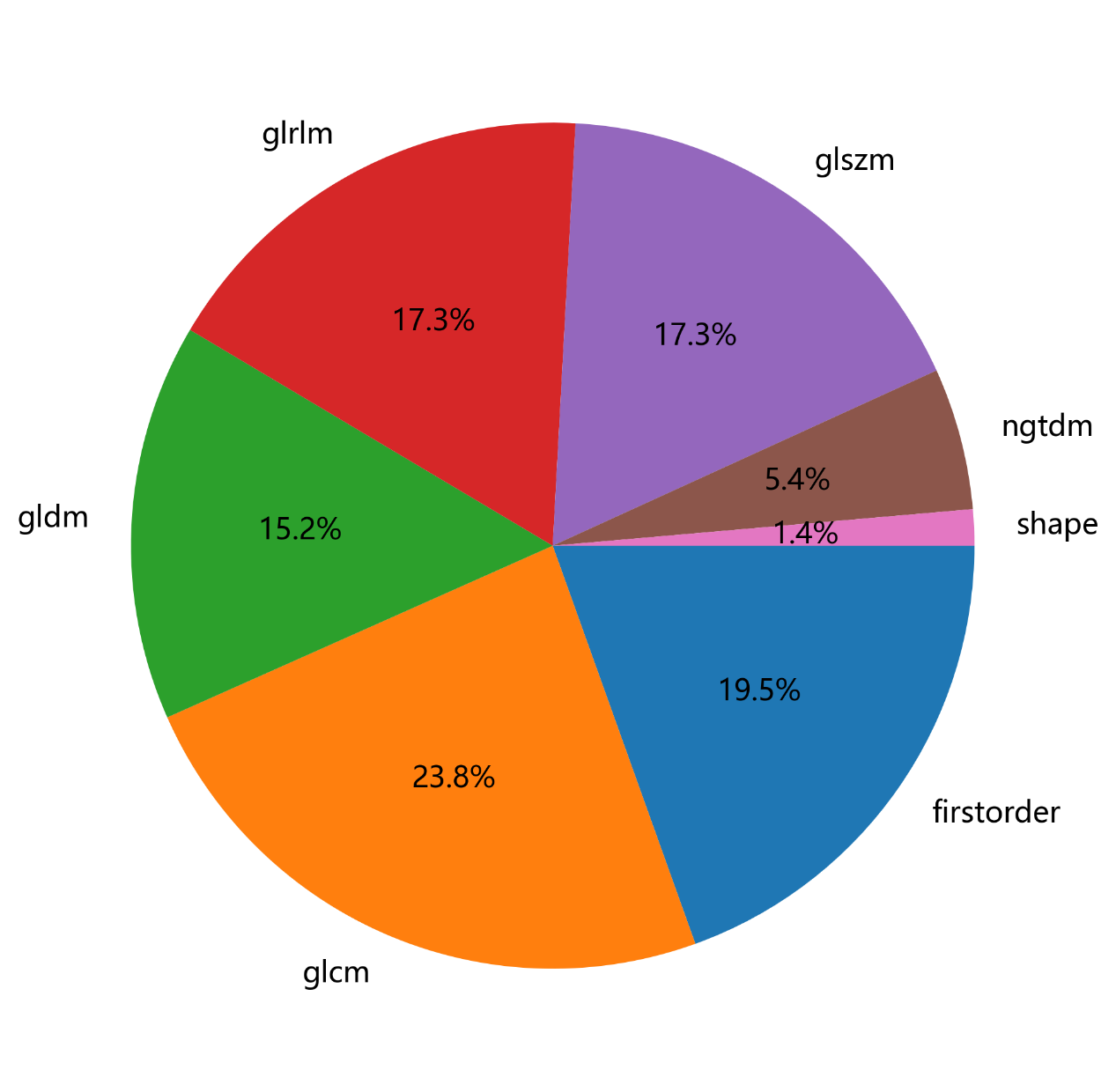


**Supplementary Fig S2:** Number and ratio of handcrafted features. The handcrafted features extracted for the CML model can be categorized into three groups: (I) geometry, (II) intensity, and (III) texture. Geometry features describe the three-dimensional shape characteristics of the tumor, while intensity features relate to the first-order statistical distribution of voxel intensities within the tumor. Texture features describe the patterns and higher-order spatial distributions of intensities. Various methods, such as gray-level co-occurrence matrix (GLCM), gray-level run length matrix (GLRLM), gray level size zone matrix (GLSZM), and neighborhood gray-tone difference matrix (NGTDM) methods, were employed to extract the texture features.


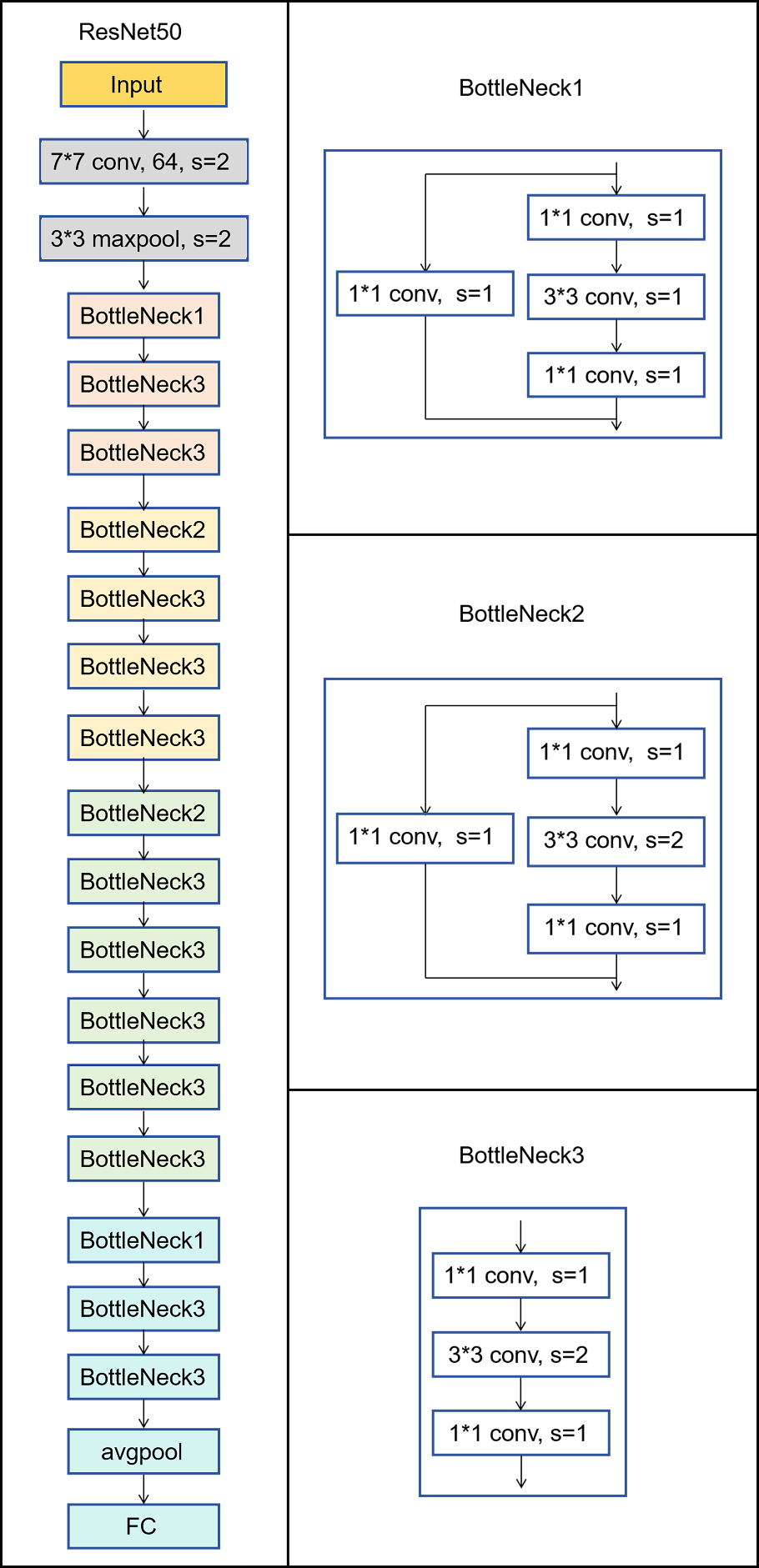


**Supplementary Fig S3:** An illustration showcasing the Resnet network architectures.


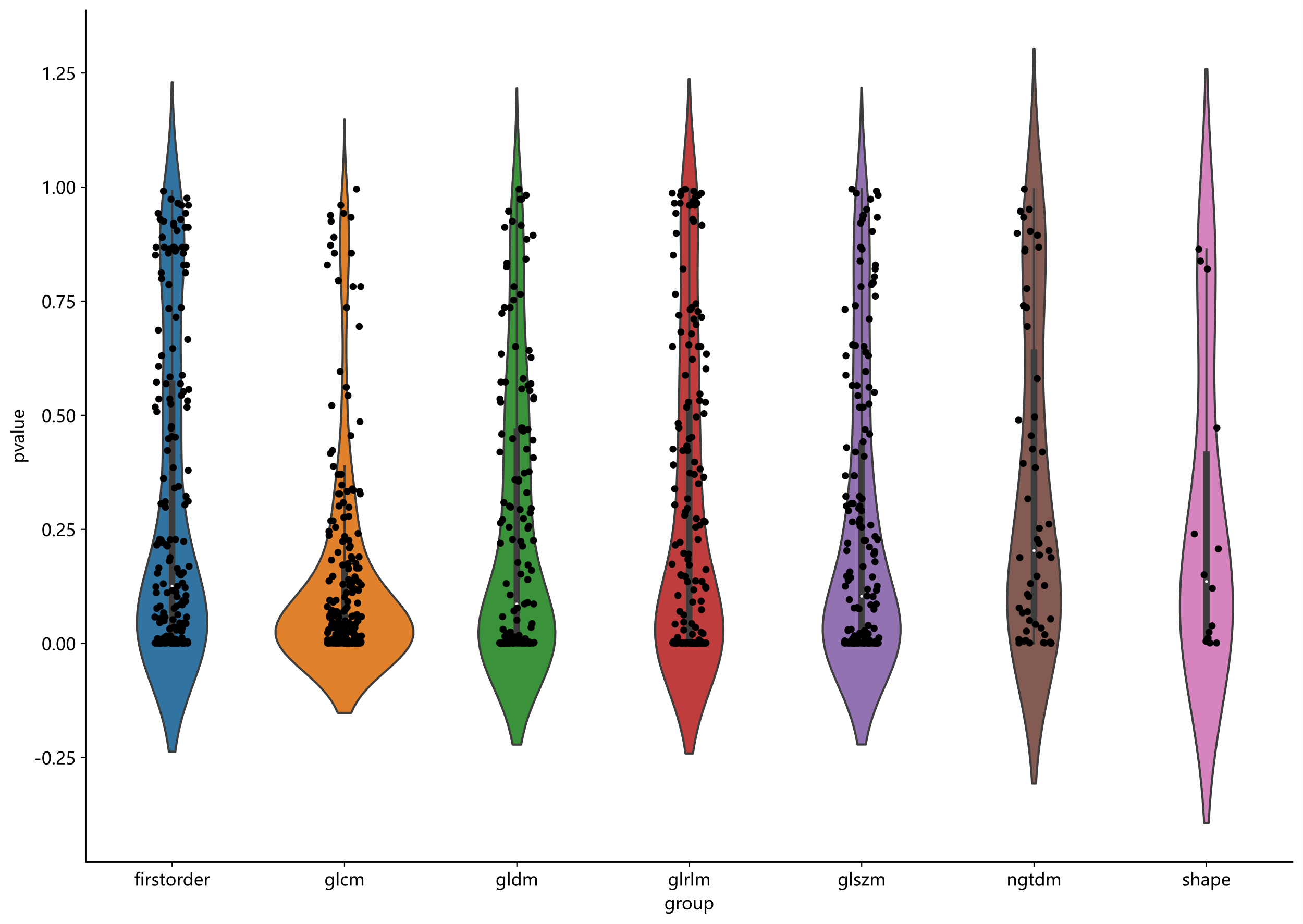


**Supplementary Fig S4:** Statistics of radiomic features.


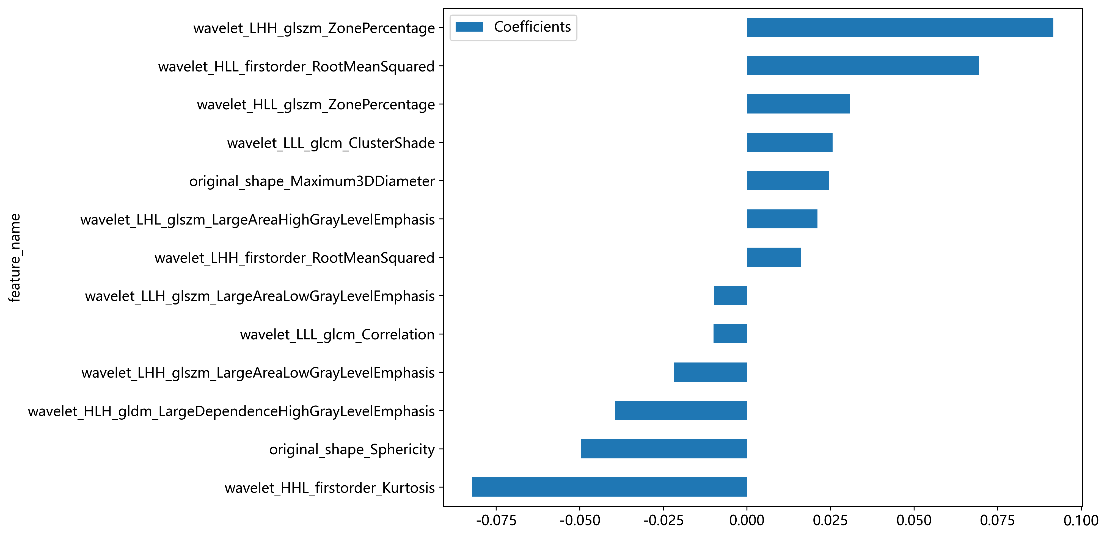


**Supplementary Fig S5:** The histogram of the Rad-score based on the selected features.


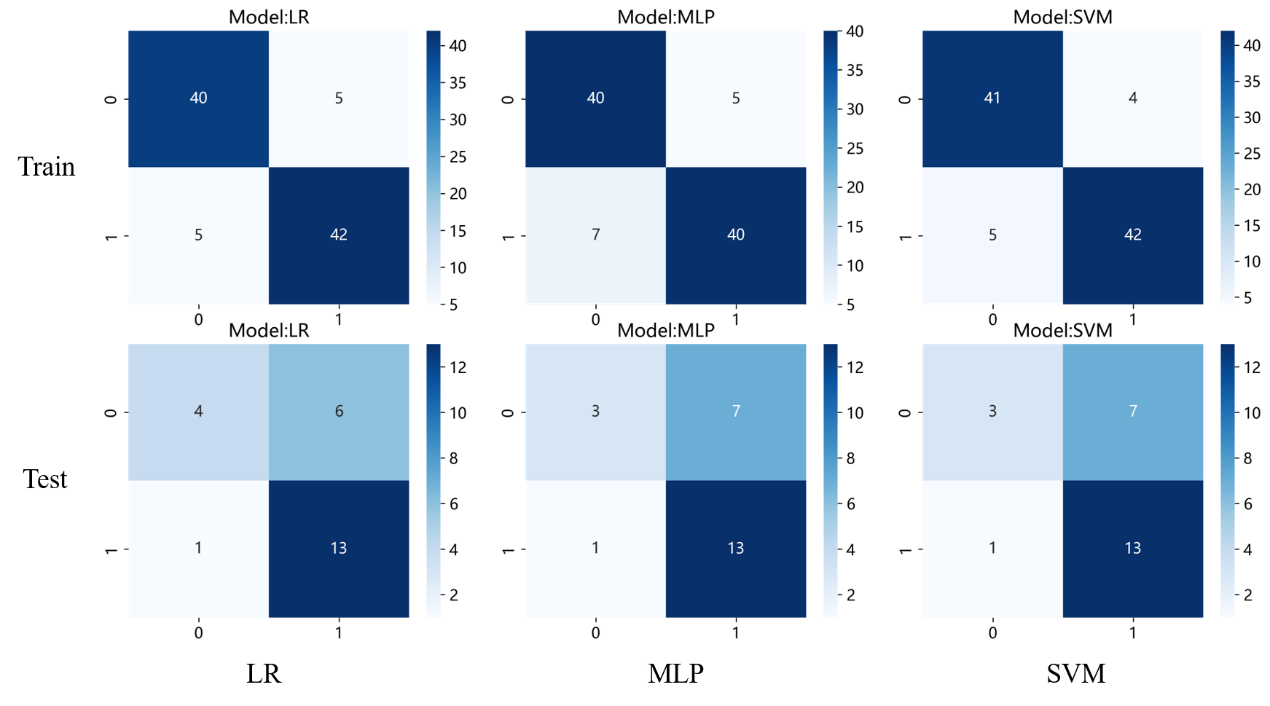


**Supplementary Fig S6a:** The confusion matrices of the prediction results on single CML model.


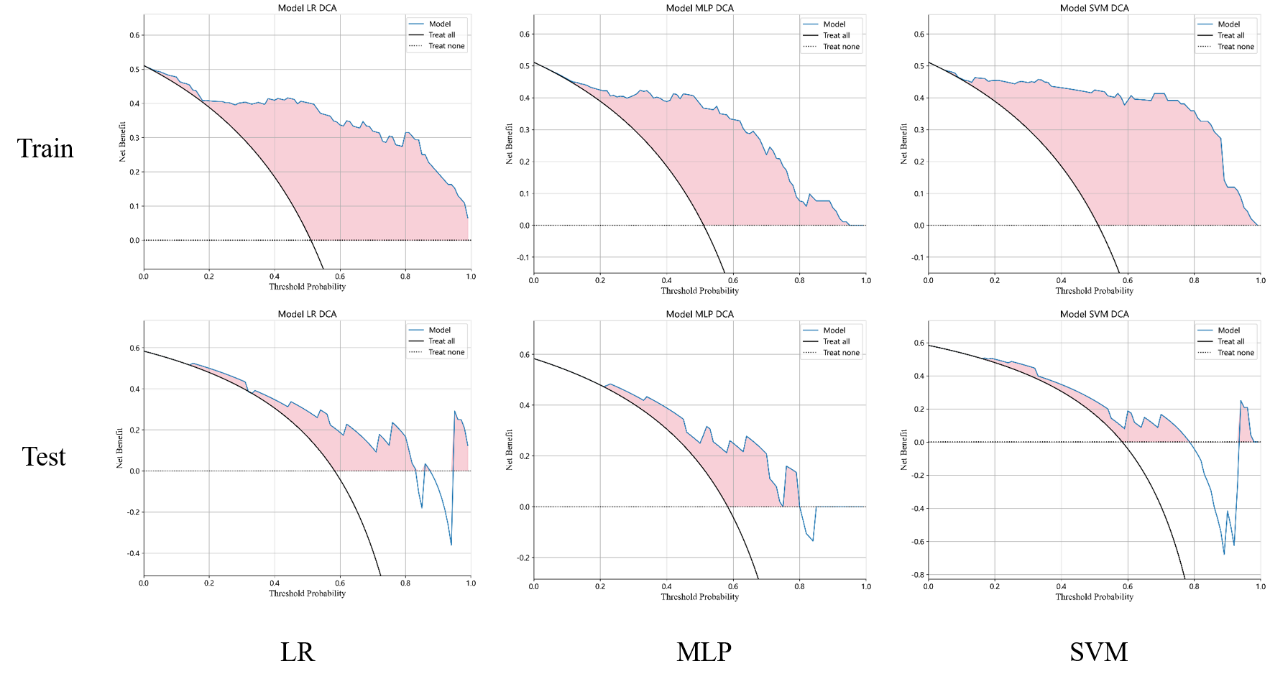


**Supplementary Fig S6b:** The DCA of single CML model.


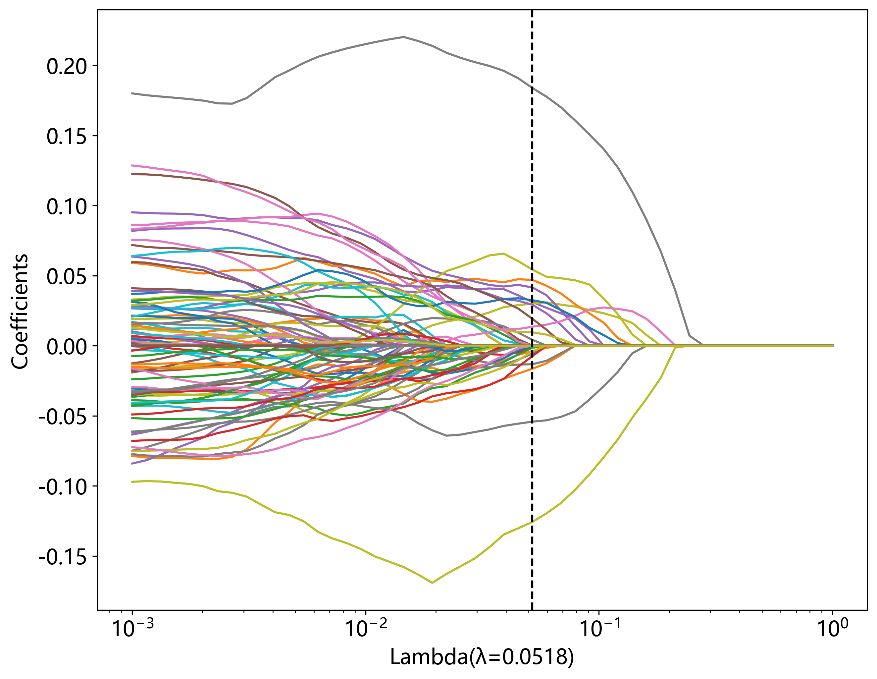


**Supplementary Fig S7a:** Coefficients of 10-fold cross validation in DLR model.


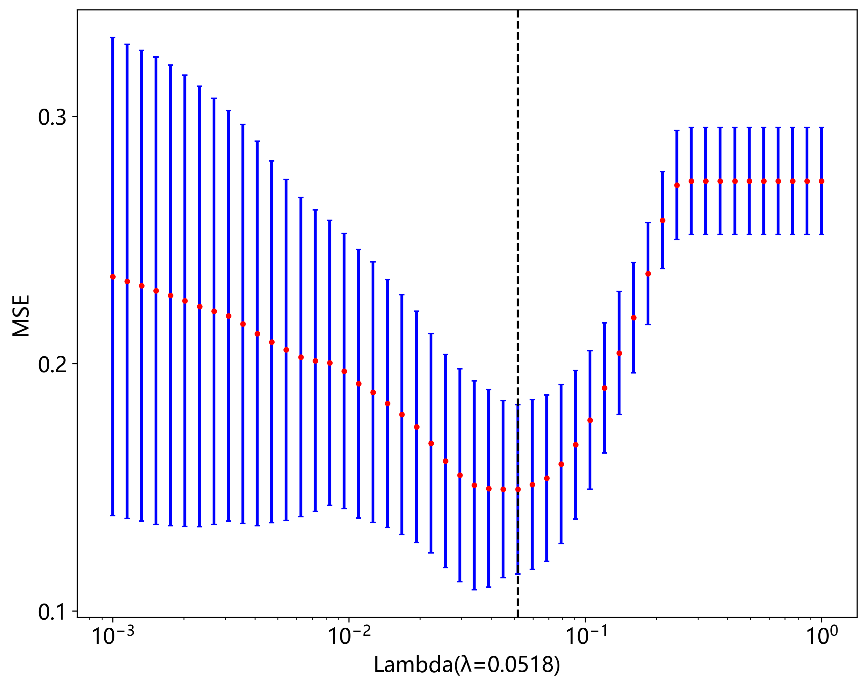


**Supplementary Fig S7b:** MSE of 10-fold cross validation in DLR model.


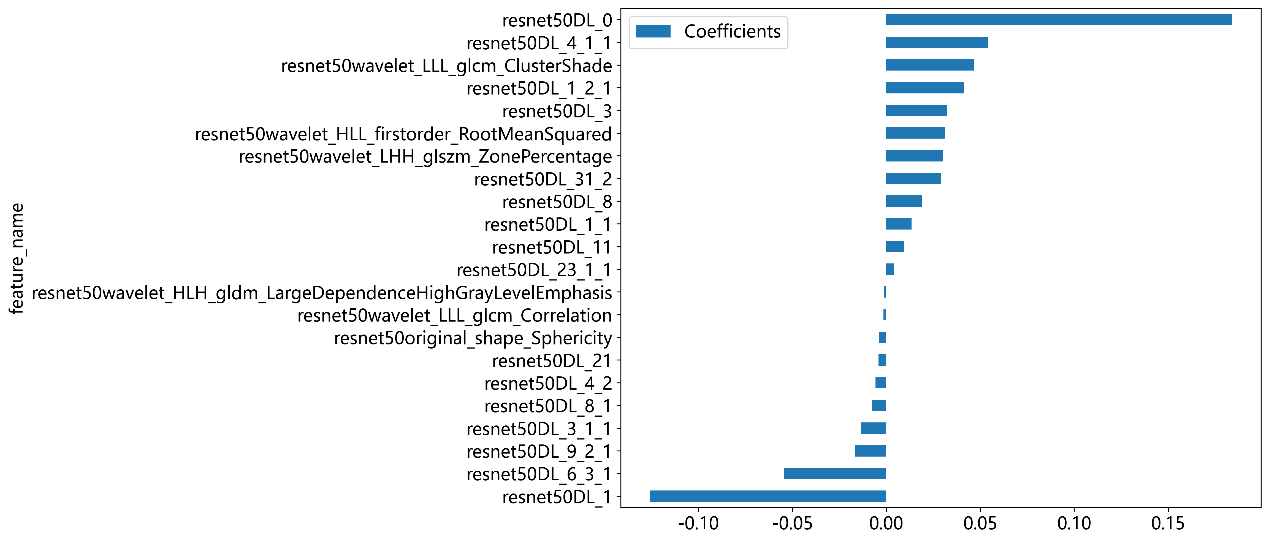


**Supplementary Fig S7c:** The histogram of the Rad-score based on the selected features in DLR model.


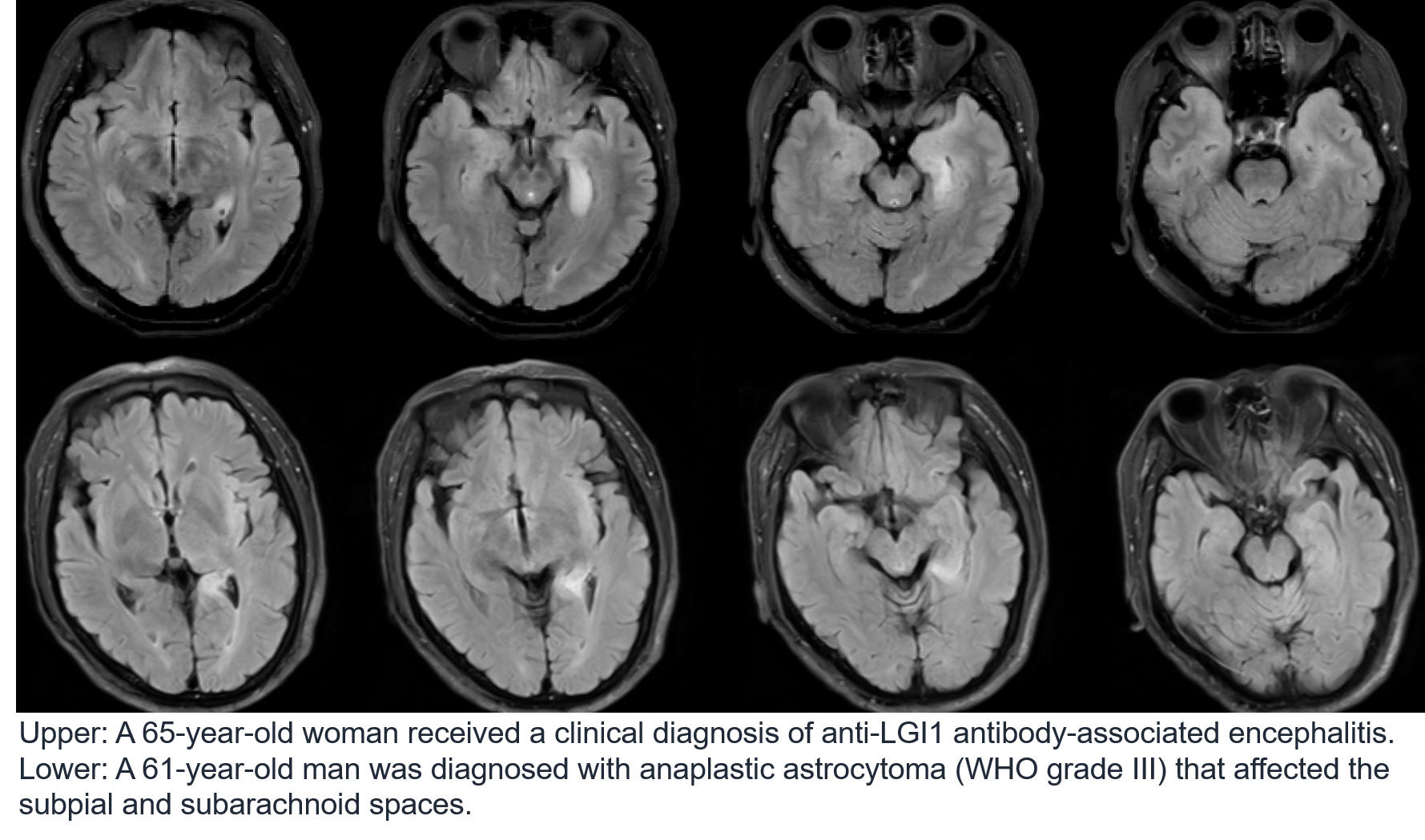


**Supplementary Fig S8:** Two characteristic examples of encephalitis and gliomas in atypical cases are presented.


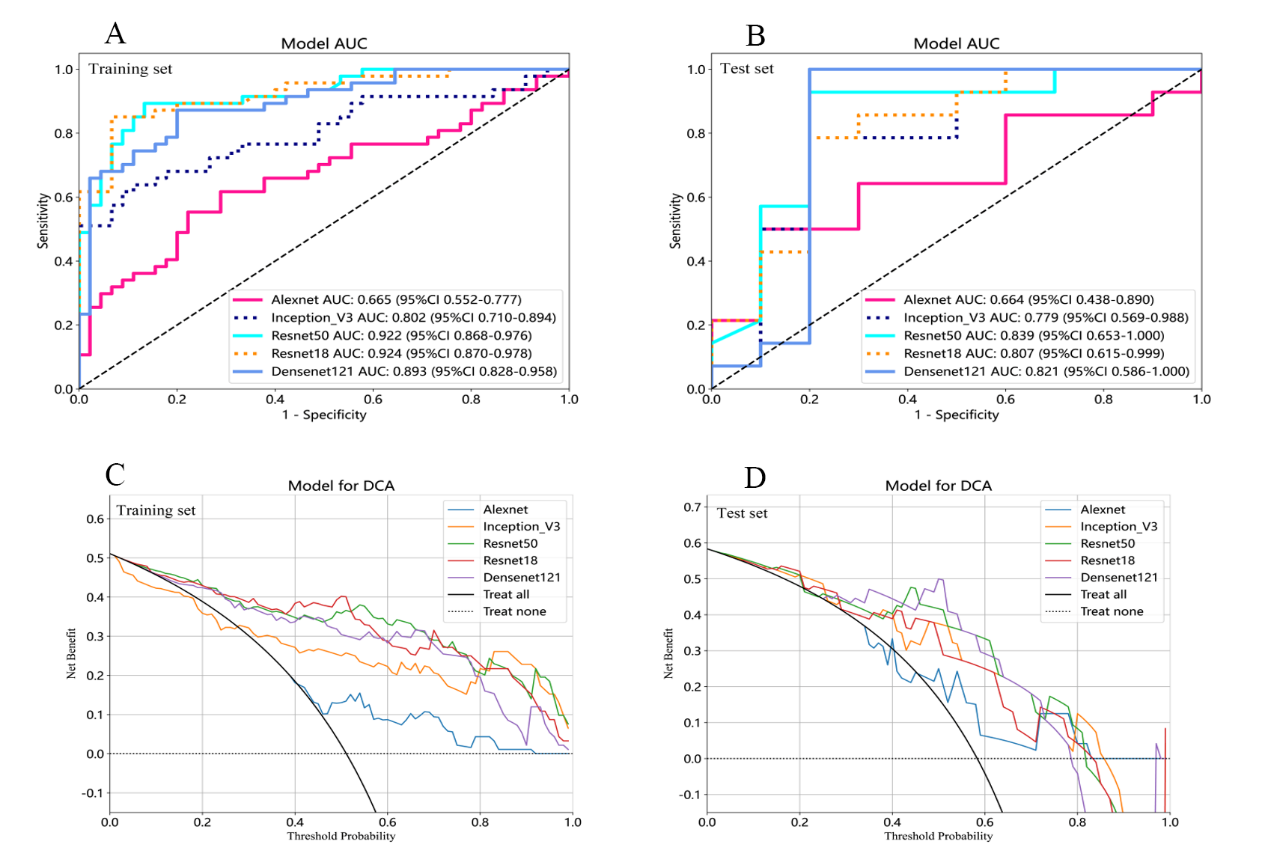


**Supplementary Fig S9: 9a, b** demonstrates ROC analysis of different DL models in the training and test sets; **9c, d** illustrates the DCA analysis of different DL models the training and test sets. The results unequivocally demonstrate that our model outperforms Alexnet and Inception-V3 in terms of performance.

**Supplementary Table 1.** The scanning parameters for the axial FLAIR sequence of each MRI scanner.

| **MRI scanner** | **TR (ms)** | **TE (ms)** | **Slice thickness (mm)** | **FOV** |
| --- | --- | --- | --- | --- |
| GE Discovery MR750 | 8000 | 148 | 5.50 | 512×512 |
| GE Medical System Genesis Signa | 9000 | 121 | 4.00 | 512×512 |
| Siemens MAGNETOM Trio TimSystem | 6800 | 81 | 5.00 | 320×320 |
| Siemens MAGNETOM Verio  GE Singa HDe | 7500  9054 | 85  138 | 5.00  5.50 | 512×464  512×512 |
| Philips Ingenia | 4800 | 255 | 5.00 | 512×512 |

**Supplementary Table 2.** The results of Delong test between the best CML, best DL model and the DLRN model in the training and validation cohort.

| Model | Training sets (*p* value) | Validation sets (p value) |
| --- | --- | --- |
| Resnet50_rad Vs Resnet50 | 0.00461101 | 0.45146933 |
| Resnet50_rad Vs Rad_LR | 0.00883335 | 0.69202068 |
| Resnet50 Vs Rad_LR | 0.83470196 | 0.97453261 |

A two-sided *p* < 0.05 was considered statistically significant.

**Supplementary Reference**

1. Rodriguez, A., et al., *LGI1 antibody encephalitis: acute treatment comparisons and outcome.* J Neurol Neurosurg Psychiatry, 2022. **93**(3): p. 309-315.
